# Supplementary material for: CRISPR-Cas9 knockout screen informs efficient reduction of the Komagataella phaffii secretome
Source: Microb Cell Fact. 2024 Jul 31;23:217. doi: 10.1186/s12934-024-02466-2 (PMC11293167; doi:10.1186/s12934-024-02466-2)
Supplement: Supplementary file 2 — Additional file 2. Supplemental figures. [file 12934_2024_2466_MOESM2_ESM.docx]

**CRISPR-Cas9 knockout screen informs efficient reduction of the *Komagataella phaffii* secretome**

**Additional File 2:** Supplemental figures

Neil C. Dalvie^1,2^, Timothy R. Lorgeree^2^, Yuchen Yang^1,2^, Sergio A. Rodriguez-Aponte^2,3^, Charles A. Whittaker^2^, Joshua A. Hinckley^2^, John J. Clark^2^, Amanda M. Del Rosario^2^, Kerry R. Love^1,2^*, J. Christopher Love^1,2^*

^1^Department of Chemical Engineering, Massachusetts Institute of Technology, Cambridge, Massachusetts 02139, United States

^2^The Koch Institute for Integrative Cancer Research, Massachusetts Institute of Technology, Cambridge, Massachusetts 01239, United States

^3^Department of Biological Engineering, Massachusetts Institute of Technology, Cambridge, Massachusetts 02139, United States

*Correspondence to: [clove@mit.edu](mailto:clove@mit.edu), [kerryluv@gmail.com](mailto:kerryluv@gmail.com)


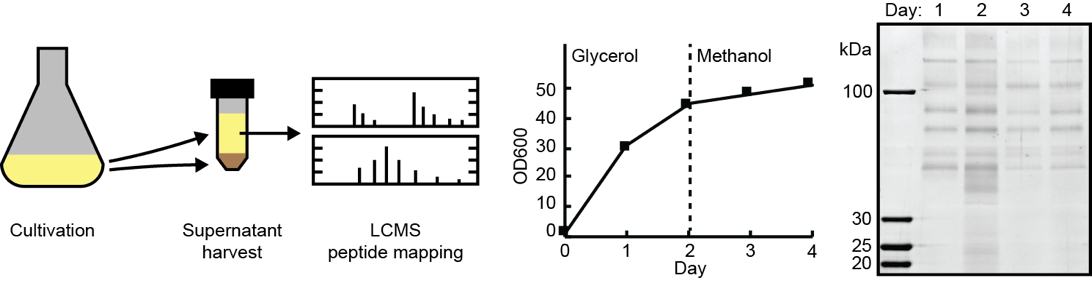


Fig. S1. Identification of secreted proteins

Cell growth and harvesting of culture supernatant (left). Representative SDS-PAGE of culture supernatant (right).


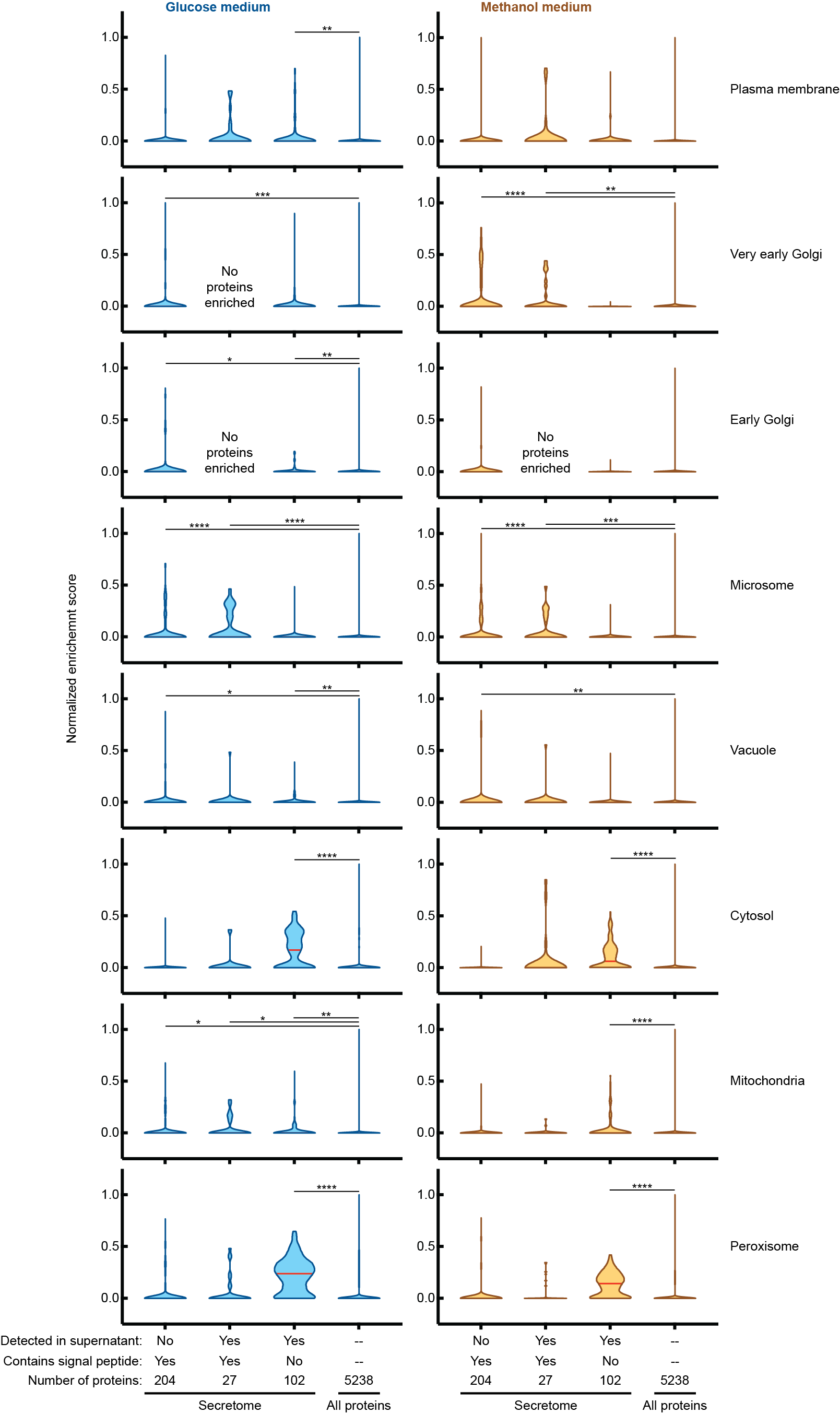


Fig. S2. Comparison of the *K. phaffii* secretome to the study by Valli et al. Proteins that were not detected by Valli et al., or that were detected but were below the cutoff, were assigned an enrichment score of 0. Red line represents the median value. Significance was determined by Kruskal-Wallis test with Dunn’s multiple hypothesis correction (p<0.05*, p<0.01**, p<0.001***, p<0.0001****).


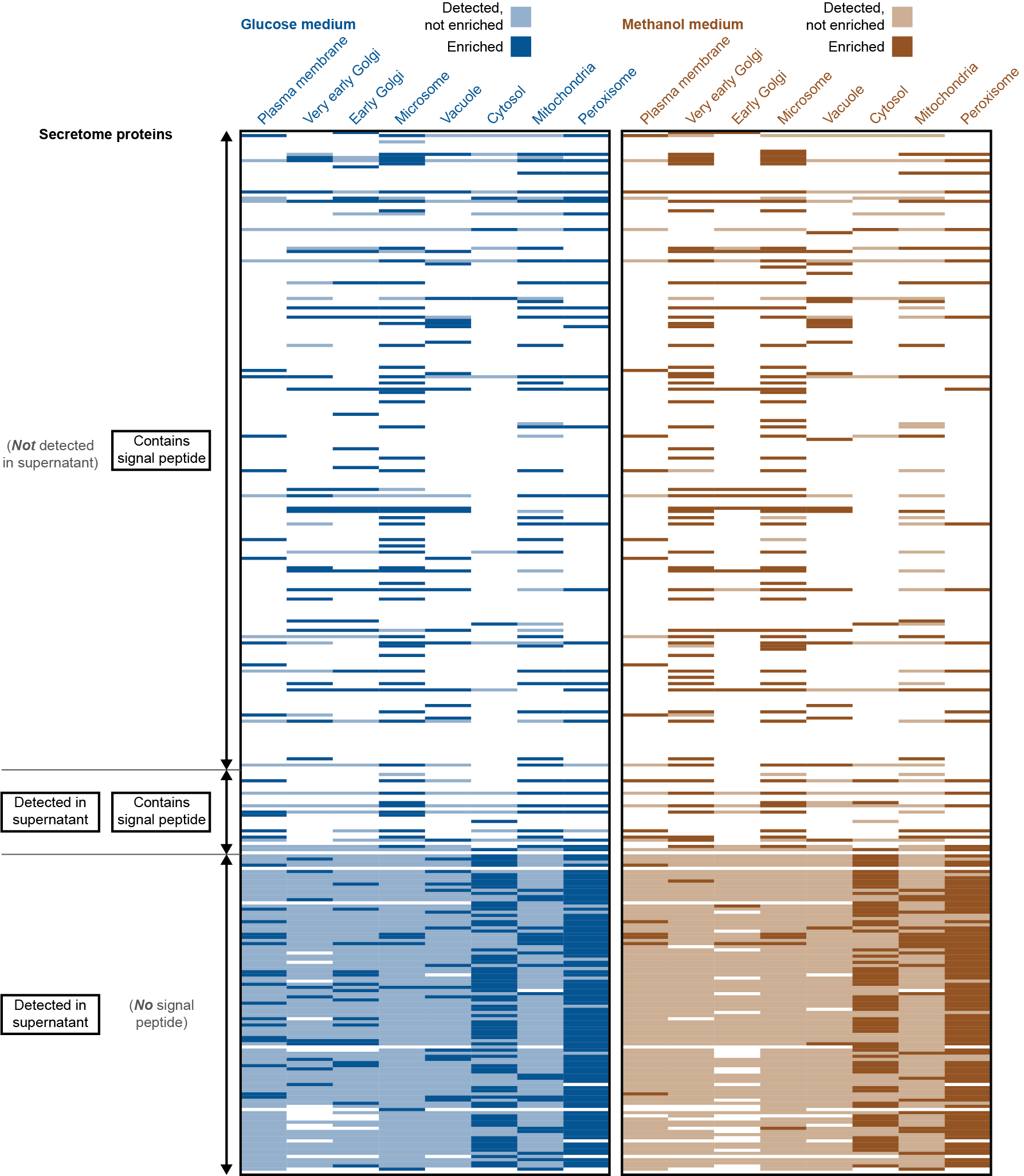


Fig. S3. Detection or enrichment of proteins in the *K. phaffii* secretome by Valli et al.


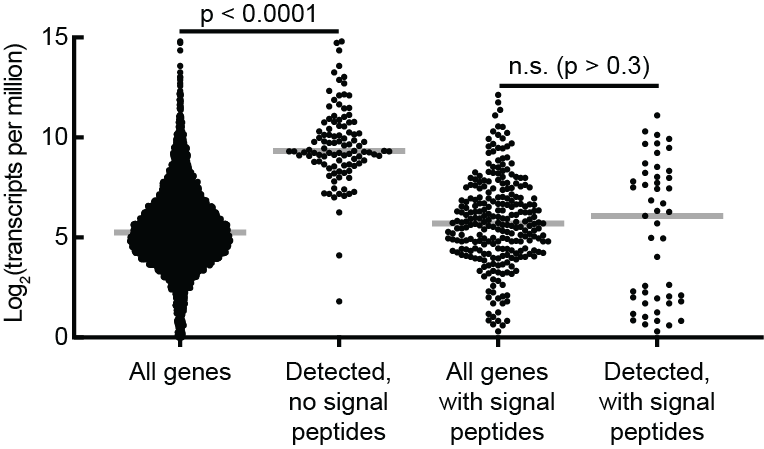


Fig. S4. Gene expression from RNAseq of an unedited base strain. Data was adapted from Love, K.R., et al. Genes labeled as “detected” code for proteins that were observed in culture supernatants by LCMS. Signal peptides were predicted by SignalP. Significance was determined by Welch’s t-test. Grey bars represent median values.


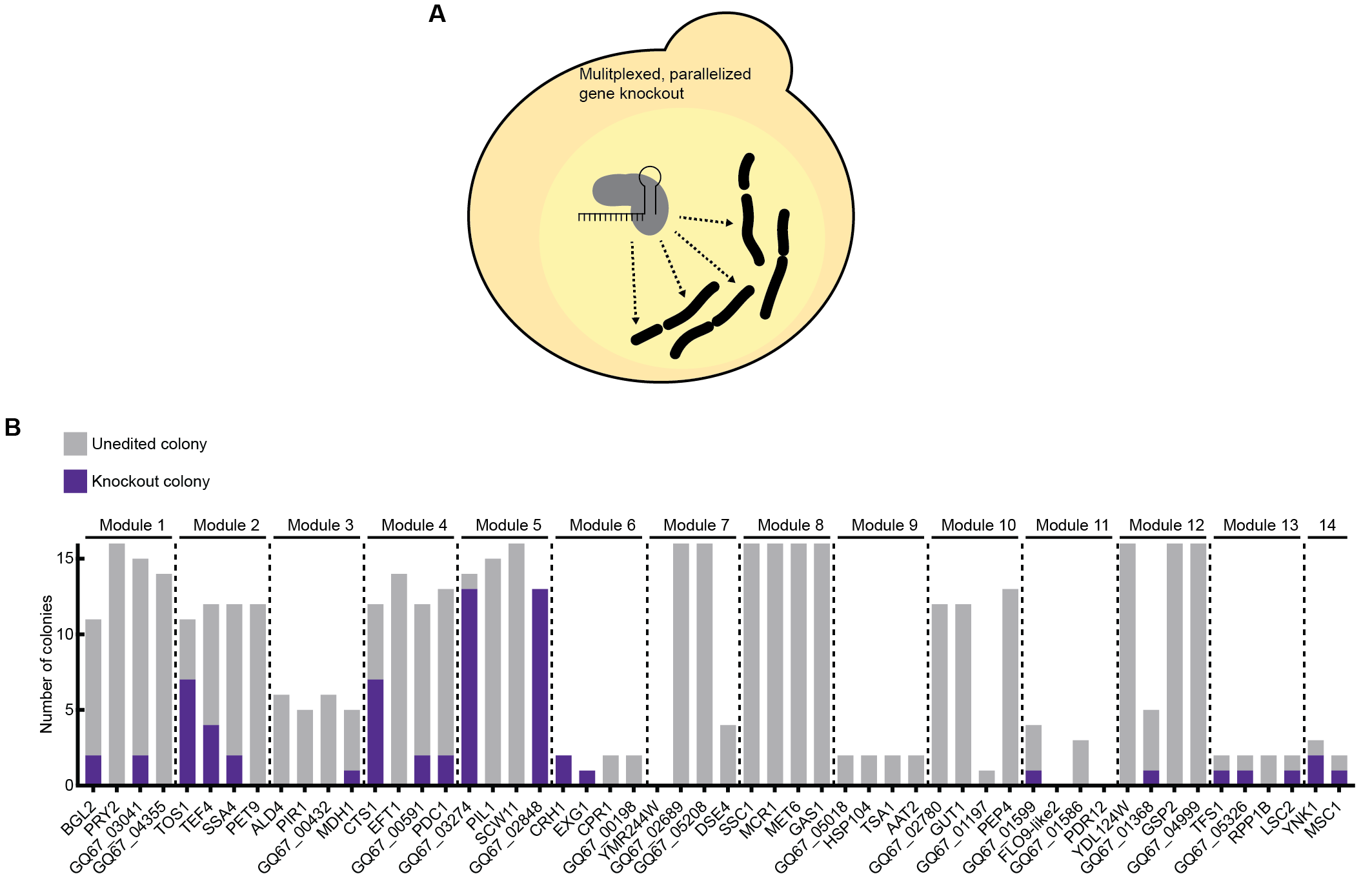


Fig. S5. Multiplexed knockout of secretome genes.

A) Schematic of simultaneous knockout of four genes. B) Results of multiplexed knockout screening. Knockout genotypes were determined by Sanger sequencing of each target locus of up to 16 colonies per transformation.


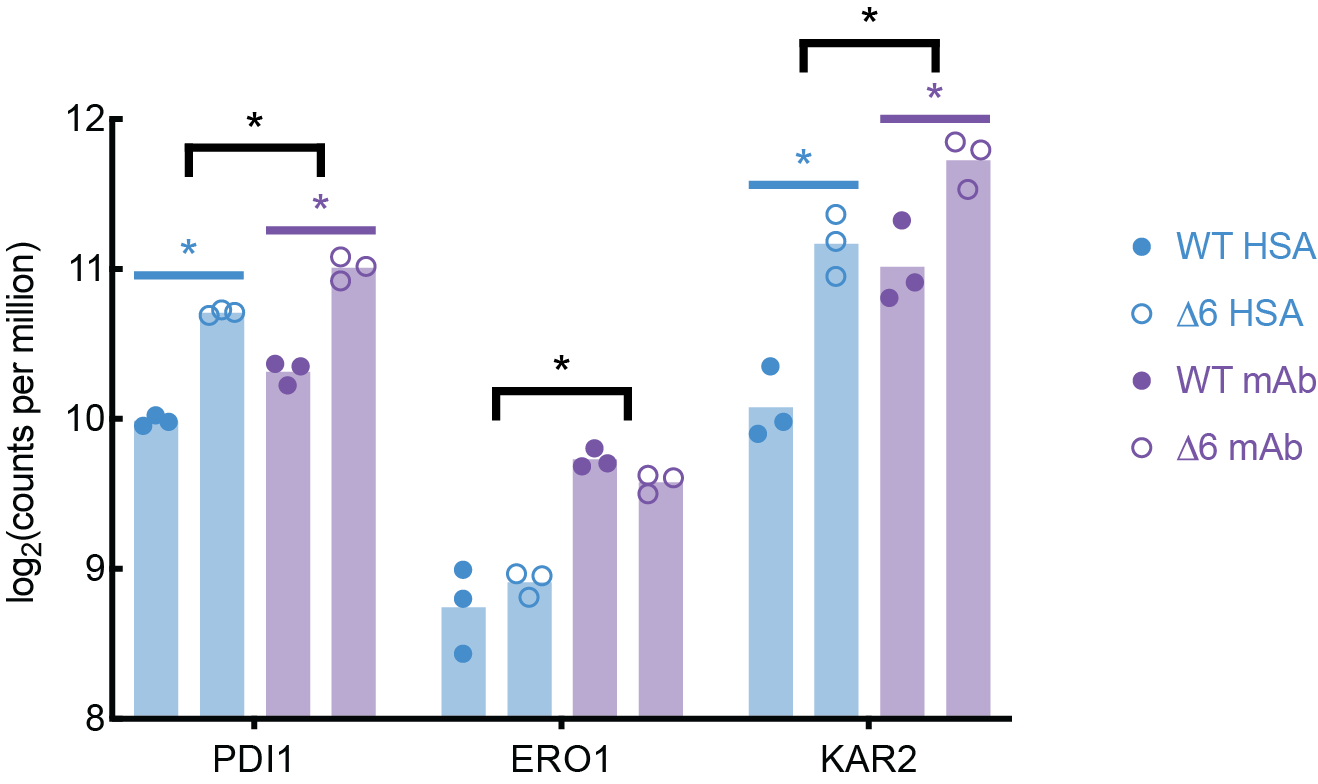


Fig. S6. Expression levels of chaperones in *K. phaffii* strains. Significance of expression differences was determined by multiple t-tests with Benjamini-Krieger-Yekutieli multiple hypothesis correction (p<0.01*).
